# PRAME Expression as Helpful Immunohistochemical Marker in Rhabdoid Melanoma

**DOI:** 10.3390/dermatopathology9020019

**Published:** 2022-05-02

**Authors:** Valerie Glutsch, Marion Wobser, Bastian Schilling, Anja Gesierich, Matthias Goebeler, Hermann Kneitz

**Affiliations:** Department of Dermatology, Venereology and Allergology, University Hospital Würzburg, 97080 Würzburg, Germany; wobser_m@ukw.de (M.W.); schilling_b@ukw.de (B.S.); gesierich_a@ukw.de (A.G.); goebeler_m1@ukw.de (M.G.); kneitz_h@ukw.de (H.K.)

**Keywords:** PRAME, rhabdoid differentiation, rhabdoid melanoma, immunohistochemistry, melanocytic markers

## Abstract

Background: Rhabdoid melanoma is a rare variant of malignant melanoma with characteristic cytomorphologic features. Due to the potential loss of conventional melanocytic markers, histopathologic diagnosis is often challenging. We hypothesize that immunostaining for *PReferentially expressed Antigen in MElanoma* (PRAME) might have the potential to uncover the melanocytic origin of these dedifferentiated tumors. Methods: Four cases of rhabdoid primary melanomas were assessed by immunohistochemistry for expression of PRAME and conventional melanocytic markers. Immunohistochemical expression patterns were analyzed in the rhabdoid primaries and, if available, associated metastases. Results: All four cases of rhabdoid primary melanomas showed a strong nuclear positivity for PRAME, while the expression of conventional melanocytic markers S100, MART-1, SOX-10 and HMB-45 was variable between the analyzed cases. Conclusions: In summary, we report four cases of rhabdoid primary melanoma with high to intermediate expression of PRAME despite the partial and variable loss of other melanocytic markers. Hence, PRAME might facilitate the recognition of this highly aggressive entity to avoid misdiagnosis due to histopathologic pitfalls.

## 1. Introduction

**Figure 1:**
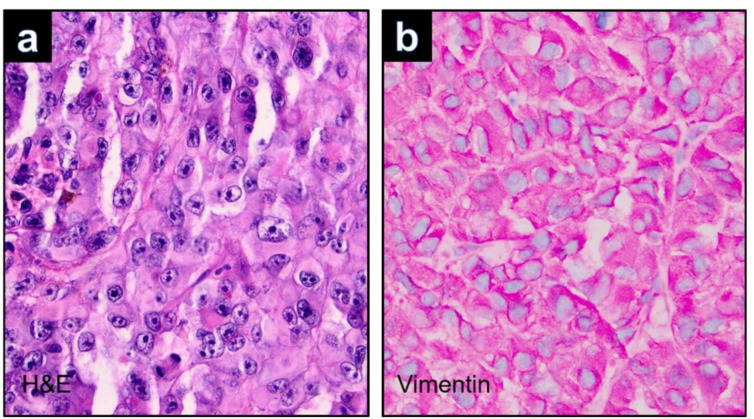
(**a**): Rhabdoid melanoma cells exhibit eosinophilic cytoplasm displaying a round eccentric vesicular nucleus with a prominent central nucleolus and large intracytoplasmic hyalin inclusions (Hematoxylin&Eosin (H&E), ×400). (**b**): Vimentin is expressed in the intracytoplasmic inclusions (Vimentin, ×400).

Malignant melanoma (MM) derives through the malignant transformation of melanocytes. Melanocytes are pigment-producing cells in human skin whose differentiation is mainly regulated by microphthalmia-associated transcription factor (MITF). Highly differentiated melanoma cells usually maintain their pigment-producing function. However, MM is also known for its ability for metaplasticity and variable differentiation [1]. First described in 1992, rhabdoid melanoma is a rare variant of MM with characteristic histopathological features including an eosinophilic cytoplasm with large hyaline inclusions and eccentric, round nuclei with prominent nucleoli [1,2] (Figure 1a). Typical immunohistochemical characteristics are the (at least partial) loss of melanocytic markers and positivity for the mesenchymal markers vimentin or desmin (Figure 1b). Most cases of rhabdoid MM described in the literature refer to the rhabdoid differentiation of metastases, while only a limited number of primary melanomas with rhabdoid histopathologic features have been published [3,4,5,6,7,8,9,10,11]. Therefore, little is known about the common histopathologic and immunohistochemical characteristics of these rare dedifferentiated primary tumors and their prognosis.

Differential diagnoses of rhabdoid melanoma include malignant peripheral nerve sheath tumor, plasmacytoma and rhabdomyosarcoma, among other rare malignancies. In general, most rhabdoid tumors stain positive for the mesenchymal markers vimentin and/or desmin [6]. Malignant peripheral nerve sheath tumors may show focal expression of S100 and SOX-10 and also of EMA (epithelial membrane antigen), synaptophysin and CD34 [12,13]. Plasmacytoma usually expresses CD138 [14], while rhabdomyosarcoma can be distinguished by the expression of muscle markers like myoglobin [4]. Immunohistochemistry thus allows for the distinction of other phenotypes and cell lineages. However, the melanocytic origin of rhabdoid primary melanomas and rhabdoid melanoma metastases is often obscured by the absence of expression of one or more conventional melanocytic markers. Since rhabdoid differentiation is associated with poor prognosis in other entities, immunohistochemical clues for an accurate diagnosis are highly desirable [15]. A relatively new marker molecule is PRAME (*PReferentially expressed Antigen in MElanoma*), an antigen that is expressed in multiple malignant tumors including MM [16]. In particular, positivity for PRAME has already shown utility in distinguishing benign melanocytic lesions from MM [17]. As the expression of PRAME has not yet been studied in rhabdoid primary melanomas, we analyzed its expression in four primary melanomas with rhabdoid differentiation.

## 2. Materials and Methods

### 2.1. Case Selection and Data Assessment

Within a retrospective setting, we retrieved four rhabdoid primary melanomas and, if available, associated melanoma metastases from our archives. Clinical data were collected from the patients’ files. Due to the retrospective nature of the study and the collection of anonymous patient data, informed consent was waived by the local Ethics Committee.

### 2.2. Immunohistochemistry

The paraffin-embedded sections were diagnosed by dermatopathologists (M.W. or H.K.). Additional immunohistochemistry for PRAME was assessed by H.K. and V.G., a resident physician with special interest in dermatopathology. Immunohistochemistry for PRAME was performed on 5 µm tissue sections of the primary tumors and, if available, associated metastases, which were deparaffinized in xylol and rehydrated in graded alcohol before. The slides were overlaid with antigen retrieval solution (Dako, Hamburg, Germany) and incubated in saturated steam for 30 min. For staining, slides were incubated with an anti-PRAME antibody (MAb EPR20330; Abcam, #219650, 1:1000) using the Dako Autostainer plus (Dako) and the Dako REAL Detection System Alkaline Phosphatase/ RED (#K5005Dako, Dako). For PRAME and melanocytic markers (S100, MART-1, SOX-10 and HMB-45), we assessed nuclear and cytoplasmatic patterns of staining. Focal positivity was marked when only some areas of the tumor stained positive.

## 3. Results

All excised tumors were diagnosed as primary cutaneous melanomas with rhabdoid differentiation based on histomorphological findings and immunohistochemistry considering S100, MART-1, SOX-10, HMB-45, vimentin and desmin. Histomorphology for patient WUE3 was equivocal, and the possibility of a melanoma metastasis with rhabdoid differentiation was discussed due to the lack of intraepithelial or junctional tumor cells or aggregates. Table 1 provides selected clinical and pathologic characteristics for all four patients. Three out of four patients were males. The median age at primary diagnosis in our cohort was 74.5 years (range 72–79). Tumor thickness was >3 mm in all patients, and three tumors had been classified as nodular melanomas (NM). The tumors were localized on the upper and lower extremity, chest or scalp.

### 3.1. Case WUE1

**Figure 2:**
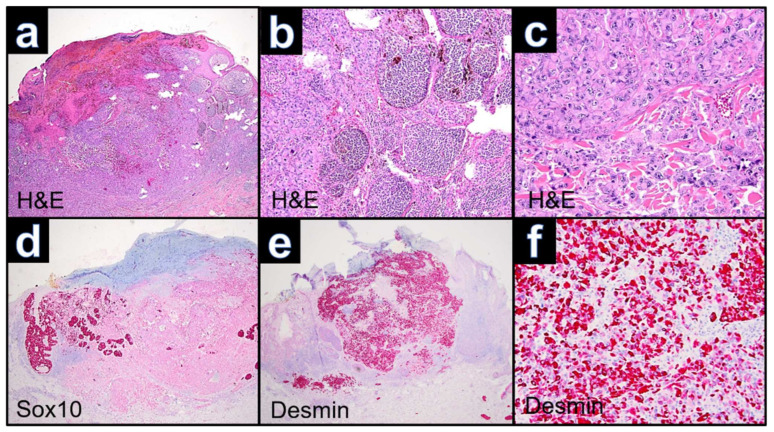
Case WUE1: (**a**): Panoramic view showing an ulcerated, relatively well-circumscribed elevated nodular lesion involving the dermis and subcutis (H&E, ×40). (**b**): Proliferation of nests or sheets of atypical polygonal neoplastic cells and nests with regular melanocytes (H&E, ×100). (**c**): Neoplastic cells with eccentrically located large round-to-oval nuclei containing conspicuous nucleoli and eosinophilic inclusions within the rich cytoplasm (H&E, ×400). (**d**): Negative staining for Sox10 of the rhabdoid differentiated melanoma cells and positivity of the regular melanocytic cells. (**e,f**): Expression of desmin by rhabdoid differentiated melanoma cells (Desmin ×40, ×100).

**Figure 3:**
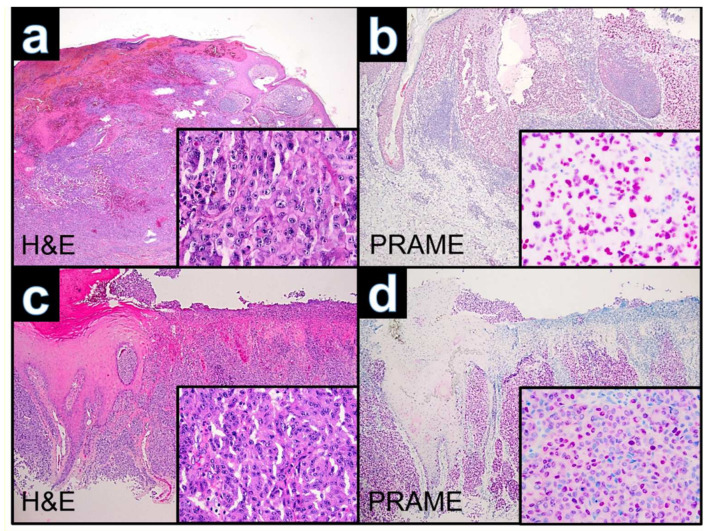
(**a**): Case WUE1: Ulcerated polypoid tumor of a 74 year old patient from the chest (H&E ×40). Inset H&E ×400. (**c**): Case WUE2: Ulcerated tumor of a 72 year old patient from the ankle (HE ×40). Inset HE ×400. (**b,d**): Cases WUE1 and WUE2: The tumor cells are diffusely positive for PRAME (nuclear labeling) (PRAME ×40). Inset highlights that PRAME labeling is nuclear (PRAME ×400).

Microscopy showed an ulcerated tumor with large complexes of atypical melanocytes in the periphery of the lesion and dedifferentiated neoplastic cells with distinct atypical nuclei and eosinophilic cytoplasm at the center of the lesion (Figure 2a–c and Figure 3a). Immunohistochemically, the tumor showed positivity for the melanocytic markers S100 (nuclear and cytoplasmatic) and MART-1 (cytoplasmatic) in the periphery, whereas both were negative in the rhabdoid differentiated area. Staining for SOX-10 (Figure 2d) and HMB-45 showed unspecific cytoplasmatic or focal cytoplasmatic positivity of the dedifferentiated neoplastic cells. Antibodies against desmin marked dedifferentiated cells (Figure 2e,f). Moreover, dedifferentiated neoplastic cells yielded a strong nuclear positivity for PRAME (Figure 3b). Mitotic activity was significantly increased. Additional immunohistochemical stains against pan-cytokeratin and CK7 were negative. One in-transit metastasis was excised and showed morphologic and immunohistochemical features in line with the rhabdoid differentiation of the primary.

### 3.2. Case WUE2

Histology revealed a superficially ulcerated nodular lesion with atypical melanocytes displaying lentiginous spurs at the lateral margins. Neoplastic cells were round with large nuclei with chromatin-rich eccentric nucleoli and eosinophilic cytoplasm (Figure 3c). Immunostaining showed positivity for the melanocytic markers S100 (cytoplasmatic), MART-1 (cytoplasmatic), SOX-10 (nuclear) and HMB-45 (cytoplasmatic). The mesenchymal marker vimentin was positive, while staining for desmin remained negative. PRAME showed a strong nuclear positivity within the neoplastic cells (Figure 3d). In addition, the immunohistochemical work-up of the primary excision revealed a dermal satellite metastasis with similar rhabdoid features.

### 3.3. Case WUE3

**Figure 4:**
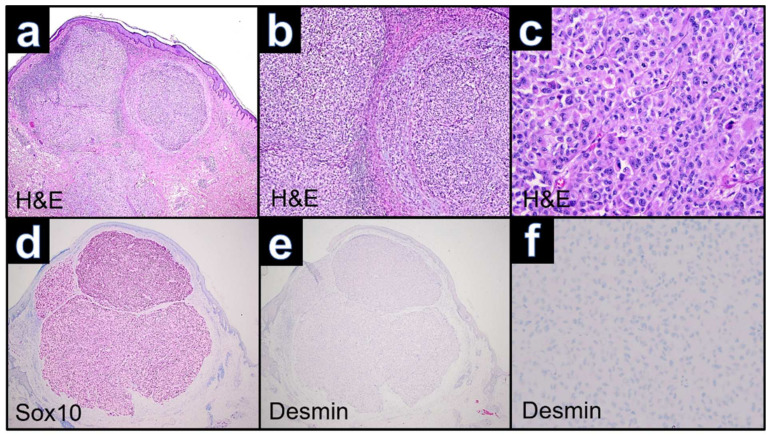
Case WUE3: (**a**): Panoramic view showing an ulcerated, relatively well-circumscribed elevated nodular lesion involving the dermis and subcutis (H&E, ×40). (**b**): Proliferation of nests or sheets of atypical polygonal neoplastic cells (H&E, ×100). (**c**): Neoplastic cells with eccentrically located large round-to-oval nuclei containing conspicuous nucleoli and eosinophilic inclusions within the rich cytoplasm (H&E, ×400). (**d**): Expression of Sox10 in rhabdoid differentiated melanoma cells. (**c**,**f**): Absence of desmin expression in rhabdoid differentiated melanoma cells (Desmin ×40, ×100).

**Figure 5:**
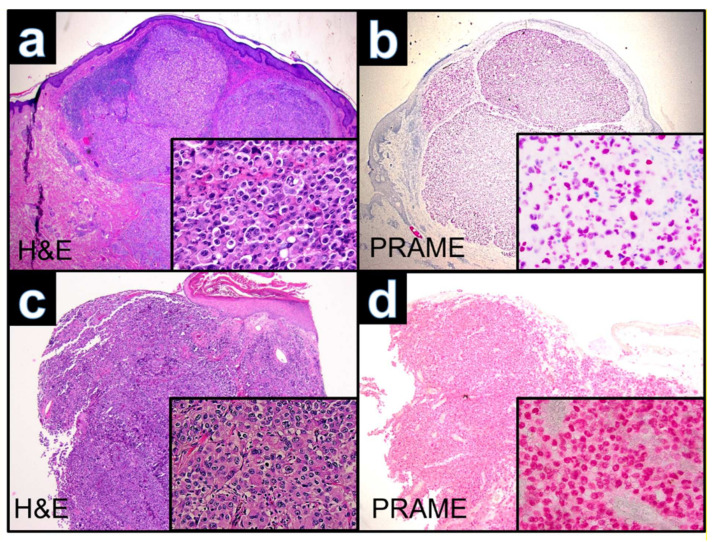
(**a**) Case WUE3: Polypoid tumor of a 79 year old patient from the arm (H&E ×40). Inset H&E ×400. (**c**): Case WUE4: Tumor of a 75 year old patient from the scalp (H&E ×40). Inset H&E ×400. (**b,d** ):Cases WUE3 and WUE4: The tumor cells are diffusely immunopositive for PRAME (nuclear labeling) (PRAME ×40). Inset highlights that PRAME labeling is nuclear (PRAME ×400).

The surgical specimen showed a dome-shaped tumor exceeding into the subcutaneous adipose tissue with a partly lobular structure. Tumor cells were large with prominent nucleoli and nuclei. However, in the lobularly structured tumor part, the neoplastic cells showed a rhabdoid differentiation with a broad eosinophilic cytoplasm and distinct pleomorphic nuclei (Figure 4a–c). Immunophenotyping revealed consistent negativity for the melanocytic markers MART-1 and HMB-45, while S100, SOX-10 (Figure 4d) and PRAME (Figure 5b) showed nuclear positivity in all tumor parts including the dedifferentiated area. Staining with vimentin was positive, whereas desmin remained negative (Figure 4e,f). Mitotic activity was very high with >20 mitoses per mm^2^. In the absence of an epidermal component, a dermal melanoma metastasis with rhabdoid differentiation was considered as the differential diagnosis.

### 3.4. Case WUE4

Microscopy of the primary tumor showed an ulcerated nodular lesion with complexes of atypical melanocytes. The latter presented with eosinophilic cytoplasm and peripheral round nuclei (Figure 5c). Immunostaining showed nuclear positivity for SOX-10, and cytoplasmatic positivity for S100, MART-1 and HMB-45. The mesenchymal marker vimentin marked the complexes of atypical melanocytes positive, while immunostaining for desmin remained negative. PRAME showed clear nuclear positivity (Figure 5d). Additional immunohistochemical stains against pan-cytokeratin and actin were negative.

## 4. Discussion

Melanoma is an aggressive cutaneous neoplasia that originates from melanocytes. Since melanocytes are pigment-producing cells in human skin, immunohistochemical melanocytic markers usually allow for an accurate histological diagnosis of these tumors. However, MM and especially melanoma metastases are known for variable and divergent histologic patterns that to some extent can mimic histopathological and immunohistochemical features of non-melanocytic neoplasms [8]. This phenotypic plasticity is linked to the neural crest origin of melanocytes [18] and hence forms the basis for the de- and transdifferentiation of MM and MM metastases.

Rhabdoid MM is a rare histopathological variant of MM with a characteristic morphologic pattern [2]. This phenomenon predominantly occurs at metastatic sites. In the literature, only ten cases of rhabdoid primary melanomas have been reported so far. Although rhabdoid differentiation is associated with poor prognosis in other malignancies, metastatic spread was described in only four of these ten cases (Table 2) [3,4,7,10]. Here, we report four additional cases of rhabdoid primary melanoma, of which three showed metastatic spread in terms of cutaneous metastases already being present at primary diagnosis (Table 1).

According to the published literature, metastasis of rhabdoid primary melanomas occurred at middle to older ages (median 68.5 years at primary diagnosis) in male patients only (Table 2). Three out of four patients already presented with regional metastases at the time of first diagnosis. Two patients were diagnosed with lymph node metastases within one month or three months after primary diagnosis, respectively. Metastases in the two remaining patients were documented after ten and 44 months. Systemic treatment for metastatic disease and outcome were reported inconsistently. In total, 2/4 patients died due to their malignant disease within six months. Since the follow-up for the published non-metastatic rhabdoid primary melanomas (*n* = 6) ranges from four to 60 months, conclusions regarding progression-free survival (PFS) and prognosis for this rare variant of MM cannot be drawn [3]. Hence, a detailed analysis and comparison of our four cases and the published metastatic cases with information on PFS and overall survival (OS) was conducted (Table 1 and Table 2). In particular, the case of a primary amelanotic rhabdoid melanoma of the forehead published by Fernández-Vega et al. has striking similarities to our case WUE1 [10]. Both patients presented with satellite or in-transit metastases at primary diagnosis and developed lymph node metastases within one month. Immunostaining of the rhabdoid areas in the primary showed positivity for vimentin and desmin. Palliative treatment was recommended. However, both patients died only two months after primary diagnosis due to rapid tumor progression. In total, 4/7 (57%) patients with metastatic rhabdoid primary melanoma died within one year after primary diagnosis, indicating a rather poor prognosis of these rhabdoid tumors.

The term “rhabdoid tumor” was also used by Haas et al. in 1981 when referring to a childhood renal cancer [19]. Since then, extrarenal tumors with rhabdoid differentiation have been reported as originating from various sites [20]. These tumors share a highly aggressive nature, which is consistent with the clinical course of the outlined patients with rhabdoid primary melanoma. Keeping this in mind, patients with rhabdoid primary melanomas might benefit from an intensified clinical follow-up and a prompt initiation of systemic therapy in the case of metastasis.

Immunohistochemically, all 10 primary tumors reported so far and our four additional cases expressed at least one melanocytic marker (Table 3). S100 stained positive in 13/14 primary tumors, while the expression of MART-1 and HMB-45 was variable. SOX-10, which is a nuclear marker like S100, stained positive in 4/5 tumors and was not studied or reported in the remaining nine primaries. In our patients, the melanoma-associated antigen PRAME provided an additional immunohistochemical clue for the diagnosis of a melanocytic neoplasia showing strong nuclear positivity in all of our rhabdoid primary melanomas (4/4, 100%), while the expression of S100, MART-1, SOX-10 and HMB-45 was variable. Immunohistochemical diagnosis of MM usually requires the expression of at least one melanocytic marker. In addition, the expression of melanogenesis-related proteins like, for example, MITF can support the diagnosis. Immunostaining for PRAME was already shown to be useful in the differential diagnosis of invasive melanocytic tumors [17]. Since the (partial) loss of conventional melanocytic markers in primary melanomas and metastases with rhabdoid differentiation makes histopathologic diagnosis challenging, additional immunostaining for PRAME could facilitate diagnosis. However, positivity for PRAME must still be interpreted with caution in tumors that are negative for other melanocytic markers, since PRAME, despite its name, is not a melanoma-specific antigen and has also shown immunoreactivity in other neoplastic entities such as non-small cell lung cancer, breast cancer, ovarian cancer and renal cell carcinoma, respectively [17]. On the other hand, positivity for conventional melanocytic markers is not probative for a melanocytic histogenesis, since few tumors of different origins can also express melanocytic markers such as clear cell sarcoma or perivascular epitheloid cell tumors (PEComa).

Rhabdoid tumors of different cell lineages are often positive for the mesenchymal markers vimentin and desmin [6,21]. However, only one of our four cases showed positivity for desmin, while vimentin stained positive in 4/4 primaries (Table 3). When reviewing the immunohistochemical profile of all reported rhabdoid primary melanomas, regardless of metastasis only two turned out to express desmin while six were negative [3,10,11]. Two primaries were not stained for desmin or staining was not reported. Staining for vimentin, however, showed positivity in 9/9 reported rhabdoid primary melanomas and was not done in one primary tumor [3,10,11]. Since this is consistent with the findings in our cohort, we conclude that positivity for desmin is not compulsory for the diagnosis of rhabdoid MM. Therefore, diagnosis of rhabdoid dedifferentiation should be made depending on typical cytomorphologic features in due consideration of immunohistochemistry.

In conclusion, our four new cases of rhabdoid primary melanoma underline possible immunohistochemical clues for diagnosis in this rare entity, namely the consistent expression of PRAME and vimentin and the irregular expression of desmin, as well as the inconsistent positivity of conventional melanocytic markers. Larger patient cohorts with longer follow-up periods are vitally important to better characterize the expression profile and prognosis of these rare tumors.

## Figures and Tables

**Table 1:** Clinical characteristics of primary rhabdoid melanomas.

Case	WUE1	WUE2	WUE3	WUE4
Age, years	74	72	79	75
Sex	male	female	male	male
MM subtype	NM	ALM	NM	NM
Location	chest	ankle	arm	scalp
Tumor thickness, mm	3.8	5	5.8	8
Metastasis at primary diagnosis	satellite metastasis	SLN and in-transit metastasis	no	in-transit metastasis
Follow-up				
Time to (further) metastasis (PFS)	3 weeks until lymph node and distant metastases	3 months until lung metastases, 6 months until brain metastases	no metastasis	no metastasis
Localization of metastases	sub-/cutaneous, lymph node, pleura	lung, brain	na	na
Treatment for metastatic disease	no	chemotherapy	na	na
OS after diagnosis	2 months	11 months		
Time of follow-up without additional metastasis	-	-	lost to follow-up	3 months

Ref., reference; MM, malignant melanoma; na, not available; NM, nodular melanoma; ALM, acrolentiginous melanoma; PFS, progression-free survival; SLN, sentinel lymph node; OS, overall survival.

**Table 2:** Clinical characteristics of reported metastatic primary rhabdoid melanomas.

Case (Ref.)	1 [4]	2 [7]	3 [3]	4 [10]
Age, years	41	74	63	80
Sex	male	male	male	male
MM subtype	na	NM	NM	na
Location	scalp	back	sole	forehead
Tumor thickness, mm	6	9	4	12
Metastasis at primary diagnosis	no	satellite metastasis	SLN metastasis	in-transit metastasis
Follow-up				
Time to (further) metastasis (PFS)	3 months until lymph node metastases	10 months to local recurrence	44 months until lung metastases	1 month until lymph node metastases
Localization of metastases	lymph node	cutaneous, lung	lymph node, lung	cutaneous, lymph node
Treatment for metastatic disease	dacarbazine, radiotherapy	na	nivolumab	radiotherapy
OS after diagnosis	6 months	na	na	2 months

Ref., reference; MM, malignant melanoma; na, not available; NM, nodular melanoma; ALM, acrolentiginous melanoma; PFS, progression-free survival; SLN, sentinel lymph node; OS, overall survival.

**Table 3:** Immunohistochemical patterns of rhabdoid differentiated primary melanomas.

Case (Ref.)	WUE1	WUE2	WUE3	WUE4	1 [4]	2 [7]	3 [3]	4 [10]	5 [5]	6 [5]	7 [5]	8 [6]	9 [8]	10 [11]
Amelanotic	no	no	no	no	na	na	yes	yes	yes	yes	yes	yes	no	yes
IHC— melanocytic markers in the rhabdoid areas of the primary														
S100	-	+	+	+	+	+	+	(+)	+	+	+	+	+	+
MART-1	-	+	-	+	+	-	+	nd	nd	nd	nd	nd	nd	nd
SOX-10	-	+	+	+	nd	nd	nd	(+)	nd	nd	nd	nd	nd	nd
HMB-45	+ (focal)	+	-	+	nd	nd	+	-	-	-	-	nd	+	-
IHC — mesenchymal markers in the rhabdoid areas of the primary														
Vimentin	+	+	+	+	nd	+	+	+	+	+	+	+	+	+
Desmin	+	-	-	-	+	nd	-	(+)	-	-	-	nd	-	-
IHC — additional staining of the primary														
PRAME	+	+	+	+	nd	nd	nd	nd	nd	nd	nd	nd	nd	nd

Ref., reference; na, not available; IHC, immunohistochemistry; nd, not determined; PRAME, preferentially expressed antigen in melanoma.

## Data Availability

Not applicable.
